# Disruption of *Aedes aegypti* Olfactory System Development through Chitosan/siRNA Nanoparticle Targeting of *semaphorin-1a*

**DOI:** 10.1371/journal.pntd.0002215

**Published:** 2013-05-16

**Authors:** Keshava Mysore, Ellen M. Flannery, Michael Tomchaney, David W. Severson, Molly Duman-Scheel

**Affiliations:** 1 Department of Medical and Molecular Genetics, Indiana University School of Medicine, South Bend, Indiana, United States of America; 2 Eck Institute for Global Health and Department of Biological Sciences, University of Notre Dame, Notre Dame, Indiana, United States of America; National Institute of Allergy and Infectious Diseases, United States of America

## Abstract

Despite the devastating impact of mosquito-borne illnesses on human health, surprisingly little is known about mosquito developmental biology, including development of the olfactory system, a tissue of vector importance. Analysis of mosquito olfactory developmental genetics has been hindered by a lack of means to target specific genes during the development of this sensory system. In this investigation, chitosan/siRNA nanoparticles were used to target *semaphorin-1a (sema1a)* during olfactory system development in the dengue and yellow fever vector mosquito *Aedes aegypti*. Immunohistochemical analyses and anterograde tracing of antennal sensory neurons, which were used to track the progression of olfactory development in this species, revealed antennal lobe defects in *sema1a* knockdown fourth instar larvae. These findings, which correlated with a larval odorant tracking behavioral phenotype, identified previously unreported roles for Sema1a in the developing insect larval olfactory system. Analysis of *sema1a* knockdown pupae also revealed a number of olfactory phenotypes, including olfactory receptor neuron targeting and projection neuron defects coincident with a collapse in the structure and shape of the antennal lobe and individual glomeruli. This study, which is to our knowledge the first functional genetic analysis of insect olfactory development outside of *D. melanogaster*, identified critical roles for Sema1a during *Ae. aegypti* larval and pupal olfactory development and advocates the use of chitosan/siRNA nanoparticles as an effective means of targeting genes during post-embryonic *Ae. aegypti* development. Use of siRNA nanoparticle methodology to understand sensory developmental genetics in mosquitoes will provide insight into the evolutionary conservation and divergence of key developmental genes which could be exploited in the development of both common and species-specific means for intervention.

## Author Summary

Although mosquitoes transmit many of the world's deadliest diseases, mosquito developmental biology has been grossly understudied. To address this issue, we have begun to develop the dengue and yellow fever vector *Aedes aegypti* as an emerging model for understanding mosquito developmental genetics. In this investigation, we analyze development of the *Aedes aegypti* olfactory system, a tissue of vector importance which is critical for recognition of human blood meal hosts and many other essential behaviors. We used small interfering RNAs (siRNAs), which were delivered through chitosan nanoparticle feedings to larvae, to target the axon guidance gene *semaphorin-1a (sema1a)*. Loss of *sema1a* resulted in a variety of larval and pupal olfactory system defects, and these defects correlated with an odorant tracking behavioral phenotype. This study revealed that *sema1a* function is required for proper olfactory system development, and these findings advocate the use of chitosan/siRNA nanoparticles as an effective means of targeting genes in *Aedes aegypti*.

## Introduction

A lack of functional genetic analyses in vector mosquitoes has prevented us from gaining insight into how the process of development is regulated in these insect vectors of human disease. To address this problem, we have begun to functionally characterize the development of the dengue and yellow fever vector mosquito *Aedes aegypti* [1]. Here, we investigate development of the *Ae. aegypti* olfactory system, a sensory system which is critical for detection of human blood meal hosts and many other vital behaviors [2]. Gross morphological descriptions of the developing *Ae. aegypti* olfactory system were described over three decades ago [3], but detailed characterization of the development of this tissue through analysis of molecular marker expression has not yet been reported. Moreover, genetic regulation of olfactory system development has not been well-characterized in mosquitoes or in any other arthropod species outside of *Drosophila melanogaster*.

Sequencing of the mosquito genomes has opened new avenues in vector biology research [4]–[6]. Recent studies have provided insight into the function of odorant receptor (OR) genes in *Ae. aegypti* [7]–[10], but genes that are essential for the proper development of the *Ae. aegypti* olfactory system have not yet been identified. Members of the Semaphorin (Sema) family of axon guidance molecules regulate the development of a number of tissues in a variety of organisms [11]. *D. melanogaster* Sema1a, a transmembrane member of this family initially studied in the context of the embryonic ventral nerve cord, is required for proper development of the *Drosophila* pupal olfactory system [12]–[15]. The convergence of *Drosophila* olfactory receptor neurons (ORNs) expressing the same receptor onto specific glomeruli, a common organizing principle in animal olfactory systems, is regulated by Sema1a [16]. Genetic mosaic analyses in *Drosophila*, in which differential levels of Sema1a expression are detected among glomeruli, demonstrated that loss of *sema1a* results in axon misprojection phenotypes in subclasses of ORNs [14], [15]. Furthermore, graded Sema1a expression in the antennal lobe is required for the proper targeting of projection neuron dendrites into the antennal lobe [13]. Although Sema1a expression has been detected in the developing *Locusta migratoria* olfactory system [17], the function of Sema1a during pupal olfactory development has not yet been assessed in insects other than *D. melanogaster*. Our recent studies have revealed differences between *D. melanogaster* and *Ae. aegypti* development, including differences in the *sema1a* loss of function embryonic nerve cord phenotypes in the two species [18]–[20]. These studies suggest that while knowledge of *D. melanogaster* development can serve as a springboard for understanding *Ae. aegypti* development, it will be critical to functionally examine the roles of developmental genes directly in mosquito pupae. Unfortunately, assessing the function of genes in insect pupae is technically very challenging, and few have attempted such experiments.

While recent studies have increased our understanding of pupal olfactory system development in *D. melanogaster* [21], [22], the genetics of larval nervous system development, and more specifically development of the larval olfactory system, is largely unexplored in most insects. The insect larval olfactory system mimics the architecture of the pupal and adult olfactory system, but is reduced in cell number and therefore less complex [23], [24]. This reduced complexity makes the larval antennal lobe an ideal tissue in which to track olfactory system development. Given that pupal olfactory system defects are known to result from loss of *sema1a* in *D. melanogaster* [13]–[15], it is possible that Sema1a may also regulate olfactory development in insect larvae. However, this hypothesis has not yet been examined in *D. melanogaster* or mosquitoes. Furthermore, early larval antennal lobe development has not been characterized in *Ae. aegypti*, and detailed characterization of this process would therefore need to precede analysis of gene function in this tissue.

The *Ae. aegypti* genome contains a single ortholog of *D. melanogaster sema1a* [19], [20], which is the focus of this investigation. It was hypothesized that this axon guidance gene would regulate neural targeting in the developing larval and pupal olfactory system of *Ae. aegypti*. Here, we use molecular marker analysis and anterograde dye fills to characterize olfactory system development in *Ae. aegypti* larvae and pupae. We then use chitosan/siRNA nanoparticle gene targeting to investigate the function of *sema1a* during *Ae. aegypti* olfactory development. The results of this investigation indicate that *Ae. aegypti* larval and pupal olfactory system development can be disrupted through chitosan/siRNA nanoparticle targeting of *sema1a*.

## Materials and Methods

### Ethics statement

This study was performed in accordance with the recommendations in the NIH Guide for the Care and Use of Laboratory Animals. Adult female mosquitoes were allowed to blood feed on anesthetized rats ∼1 week after emergence. The protocol for maintenance and care of experimental animals including blood feeding of mosquitoes (Study # 11-036) was approved by the Institutional Animal Care and Use Committee at the University of Notre Dame.

### Mosquito rearing

The *Aedes aegypti* Liverpool-IB12 (LVP-IB12) strain was used in these investigations and reared as previously described [25].

### siRNA-nanoparticle-mediated knockdown

Knockdown of *sema1a* was achieved via chitosan/siRNA-nanoparticle feedings. Both the control and siRNAs targeting *sema1a* (siRNA^890^ and siRNA^1198^) that were utilized in this investigation have been described previously [20]. These siRNAs were used in the preparation of chitosan/siRNA nanoparticles using an adaptation of the procedure described by Zhang *et al.* [26], who prepared nanoparticles with dsRNA that were fed to *Anopheles gambiae*. *Ae. aegypti* larvae were fed on the nanoparticles for four hour time periods daily for three days (1 pellet/feeding/50 larvae). For all specific phenotypes assessed, two or three replicate experiments (n = 100 per control or experimental group) were performed with siRNA^890^, siRNA^1198^, or a combination of siRNA^890^ and siRNA^1198^. Knockdown was confirmed through *in situ* hybridization and antibody staining (see details below), as well as through qRT-PCR. For the qRT-PCR assays, five biological replicates, each consisting of at least 10 pooled control vs. experimental L4 animals, were performed and analyzed as previously described [20].

### Staining and imaging

Immunohistochemical staining was carried out as described [27], [28]. anti-HRP conjugated to Cy3 (1∶100; Jackson Immunoresearch, West Grove, PA) was used to view axon trajectories. mAb nc82 (1∶50; Developmental Studies Hybridoma Bank, Iowa) was used to visualize the synaptic neuropil. Rat anti-5HT (1∶100; Abcam, Cambridge, MA) was used to mark serotonergic projection neurons. Rabbit anti-Sema1a (1∶1000; kindly provided by A. Kolodkin) was used for analysis of Sema1a protein expression. This antibody [29] was generated against an extracellular region of *D. melanogaster* Sema1a that is conserved in *Ae. aegypti*. The antibody, which was recently used to detect Sema1a expression in grasshoppers [17], was found to specifically recognize *Aae* Sema1a in the developing brain, as assayed by the lack of brain staining assessed in *Ae. aegypti sema1a* knockdown animals (see below). Secondary antibodies goat anti-mouse FITC, goat anti-mouse Cy3, goat anti-rabbit FITC (Jackson Immunoresearch, West Grove, PA), and Alexa Fluor 568 goat anti-rat IgG (Life Technologies, Grand Island, NY) were used at a concentration of 1∶200. Brains were imaged with a Zeiss 710 confocal microscope using Zen software. Scanned images were analyzed using FIJI and Adobe Photoshop software.

For *in situ* hybridization, digoxygenin-labeled antisense and sense control riboprobes were synthesized according to the Patel [30] protocol for *sema1a* (AAEL002653). *In situ* hybridization was performed as previously described [31]. Samples were imaged on a Zeiss Brightfield microscope using AxioVision software.

### Anterograde tracing of antennal sensory neurons

Sensory neurons of fourth instar and 24 hour APF pupal antennae were anterogradely traced through the application of dextran tetramethylrhodamine (D7162, Life Technologies, Grand Island, NY) as described [32], [33]. The brains were subsequently dissected and colabeled for expression of other markers as previously discussed [28].

### Behavioral assay

Individual *Ae. aegypti* fourth instar larvae that had been fed with control or *sema1a* knockdown chitosan/siRNA nanoparticles were tested in behavioral assays that were performed generally as described by Liu *et al.* [34]. In the assay, a yeast odorant pellet was placed on one side of a petri dish, and individual larvae were placed at the opposite end of the petri dish. Individuals were scored for touching (score = 1) or not touching (score = 0) the yeast during a five minute assay. Data from four replicate experiments were compiled for statistical analysis which was completed using the Student's t-test. Following the behavioral assay, levels of *sema1a* were assessed through *in situ* hybridizaton in control vs. *sema1a* knockdown animals that had touched or not touched the yeast.

### Accession numbers

*Ae aegypti sema1a* corresponds to Vectorbase AAEL002653 and NCBI Reference Sequence: XP_001661952.1.

## Results

### *Ae. aegypti* antennal lobe development

**Figure 1:**
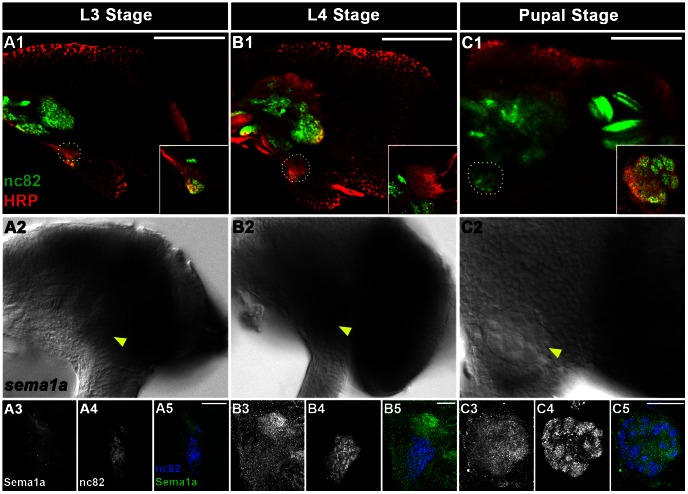
*sema1a* expression correlates with antennal lobe development in *Ae.* aegypti. The presumptive antennal lobe (circles in A1–C1; one brain hemisphere shown) is marked by HRP and monoclonal antibody mAb nc82 staining in the region just ventral to the SuEG in L3 (A1), L4 (B1) and 24 hours APF (C1) animals. Insets in A1–C1 show high magnification images (and slightly different focal planes) of the same antennal lobes co-stained for HRP and mAb nc82. Individual glomeruli are visible at 24 hours APF (marked by mAb nc82 stain in C1). Expression of *sema1a* transcript (A2, B2, C2) and protein (A3, B3, C3) is detected in larval and pupal brains (A2, A5 = L3; B2, B5 = L4; C2, C5 = 24 hours APF). Arrowheads mark the antennal lobes in A2, B2, and C2, and high magnification views of antennal lobes (co-stained for Sema1a and mAb nc82) are shown in A3–A5 (L3), B3–B5 (L4), and C3–C5 (24 hours APF). Brains are oriented dorsal up in all panels. Scale bar = 100 µm in A1, B1, and C1 and 25 µm in A3–A5, B3–B5, and C3–C5.

Application of cross-reactive antibodies has enabled visualization of components of the developing brain of *Ae. aegypti* [28]. These antibodies were used to examine olfactory development in *Ae. aegypti* larvae. The presumptive antennal lobe is visualized as a dense region marked by staining with a pan-neuronal antibody (anti-HRP) in third instar larvae (L3, Fig. 1A.1). At this time, monoclonal antibody (mAb) nc82 labeling of the synaptic neuropil marker Bruchpilot initiates (Fig. 1A.1). By the late fourth instar larval (L4) stage, mAb nc82 detects the antennal lobe as a distinct neuropil (Fig. 1B.1) and marks the region targeted by antennal ORNs (see below). At the mid-pupal stage (24 hours after puparium formation, APF, Fig. 1C.1) the antennal lobe is morphologically distinct, suggesting that the ORNs have targeted (starting from 9–12 h APF) to their respective glomeruli within the antennal lobe and synapsed with second order projection neurons. From this stage (24 hours APF) until emergence as adults, morphological examinations [28] have suggested that the antennal lobe undergoes structural refinement and fine-tuning of these established connections.

### *sema1a* expression and chitosan/siRNA nanoparticle-mediated knockdown of *sema1a* in the developing *Ae. aegypti* olfactory system

**Figure 2:**
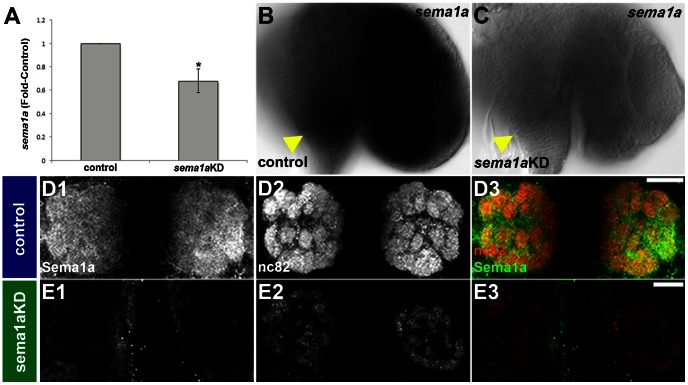
Chitosan/siRNA nanoparticles elicit knockdown in the developing olfactory system of *Ae.* aegypti. Knockdown of *sema1a* was assessed in mosquitoes fed with nanoparticles containing a mixture of *sema1a*-targeting siRNA^1198^ and siRNA^890^ (A, C, E). qRT-PCR experiments (A) demonstrated that *sema1a* levels were significantly reduced in whole L4 knockdown (KD) animals as compared to control-fed animals (p<0.01, N = 5, where N is the number of biological replicates). Nearly complete loss of *sema1a* transcript expression is observed in the brain of an L4 animal fed with chitosan/siRNA nanoparticles targeting *sema1a* (C; compare to control-fed animal in B). Arrowheads mark the antennal lobes in B and C, and brains are oriented dorsal up in both panels. Knockdown could also be detected through anti-Sema1a antibody staining 24 hours APF (E1). In control-fed pupae, Sema1a protein (D1) is detected in a wild-type lateral to medial gradient expression pattern 24 hours APF. The nearly complete loss of Sema1a expression observed in an animal fed with chitosan/siRNA nanoparticles targeting *sema1a* results in glomeruli malformation (visualized with mAb nc82 staining in E2, levels are increased to view glomerular structures; compare to control-fed animals in D2). Merge images are shown in D3 and E3. Images in D and E are a compilation of 5 confocal sections so as to ensure that the gradient is not an artifact of natural tissue curvature. Dorsal is up in all panels. Scale bar = 25 µm.

**Figure 3:**
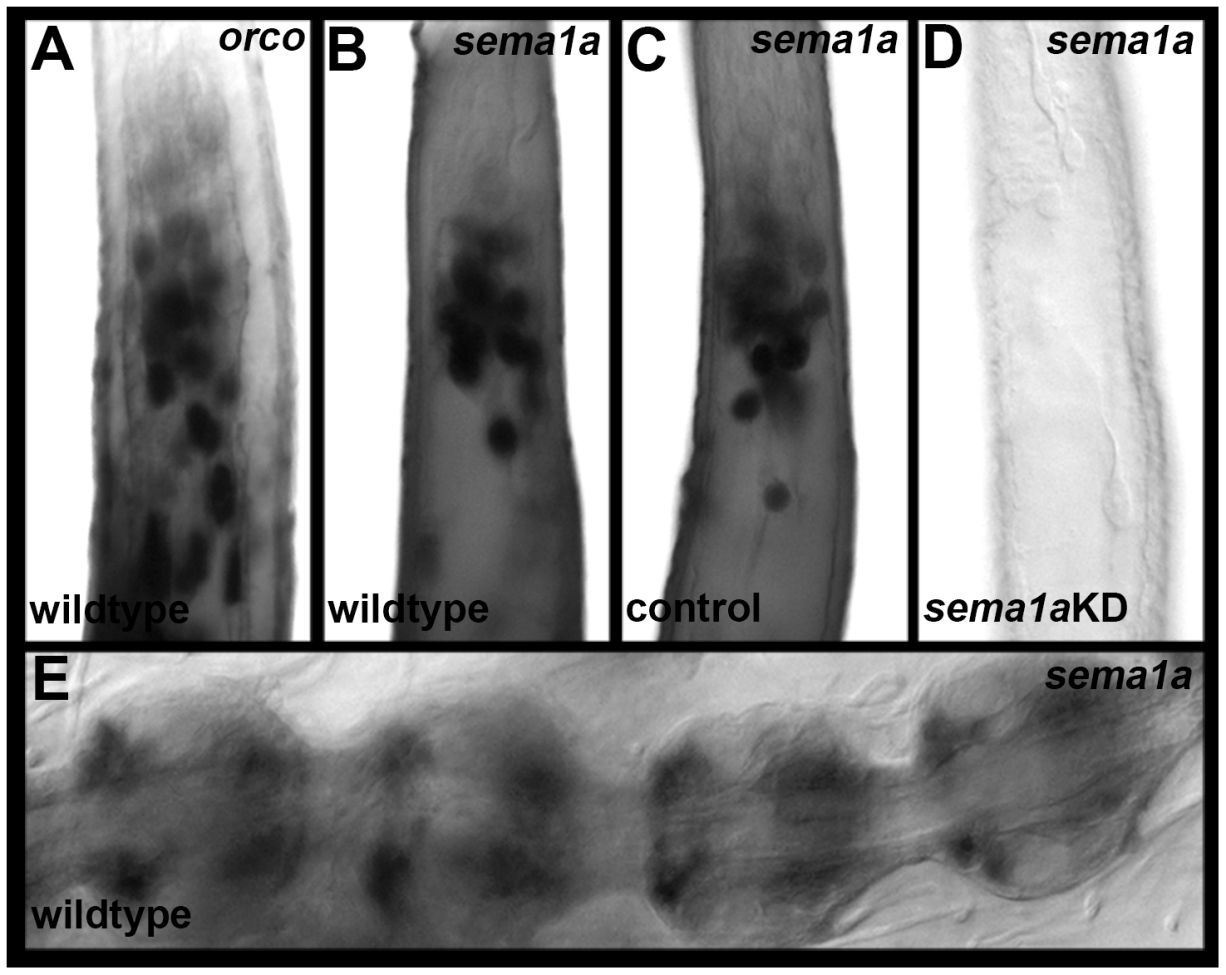
*sema1a* transcript is expressed in the antennae of larvae and pupae. Antennal ORNs, a subset of which express *sema1a* transcripts (B), are marked by *orco/OR7* transcript expression (A) in the antennae of fourth instar larvae. *sema1a* transcript expression is maintained in the wildtype pupal antenna at 24 hours APF (E). In contrast to L4 animals in which *sema1a* transcript expression is not altered following feeding with control siRNA/chitosan nanoparticles (C), no *sema1a* transcript expression is detected in L4 ORNs of an animal fed with nanoparticles containing a mixture of *sema1a*-targeting siRNA^1198^ and siRNA^890^ (D). The proximal ends of antennae are oriented up in A–D and right in E.

*sema1a* transcript expression initiates in the presumptive antennal lobe in L3 larvae (Fig. 1A.2), where it persists throughout L4 (Fig. 1B.2) and begins to diminish by 24 hours APF (Fig. 1C.2). Sema1a protein expression initiates in the L3 antennal lobe (Fig. 1A.3), where it persists during the L4 stage (Fig. 1B.3). Sema1a expression is detected in a lateral to medial gradient in the pupal antennal lobe (Fig. 1C.3, Fig. 2D.1). *sema1a* expression is also found in L4 antennal neuron cell bodies (Fig. 3B), including ORNs, 12 of which were identified by *olfactory co-receptor* (*orco*, also known as *OR7*) transcript expression (Fig. 3A). *sema1a* transcript expression is also detected in pupal antennal sensory neurons (Fig. 3E).

Following confirmation of *sema1a* expression within the developing olfactory system, a combination of two siRNAs (siRNA^890^ and siRNA^1198^) that were previously microinjected into embryos to target *sema1a* [20] were delivered to larvae via chitosan/nanoparticles. siRNA nanoparticles containing a scrambled sequence lacking homology to any known *Ae. aegypti* gene [20] served as the control in all experiments. qRT-PCR assays (Fig. 2A) with pooled whole animals indicated that in comparison to control-nanoparticle fed animals, *sema1a* knockdown animals had on average a 32+/−10% reduction in *sema1a* transcripts (p = 0.01). Chitosan/siRNA nanoparticle-mediated knockdown of *sema1a* was evident in the developing olfactory system at both the L4 (Figs. 2C vs. B; 3D vs. C) and pupal stages (Fig. 2E.1 vs. D.1), the times at which phenotypes were subsequently assayed. As was the case with microinjected embryos [20], nearly complete knockdown was detected through *in situ* hybridization/anti-Sema1a antibody staining in the developing olfactory systems of a subset of these larvae and pupae (Figs. 2C,E.1, 3D). This permitted phenotype analyses (see below) in animals that were essentially the equivalent of conditional *sema1a* null mutants and which could be identified using immunohistochemical methods.

### *sema1a* is required for larval antennal lobe development

**Figure 4:**
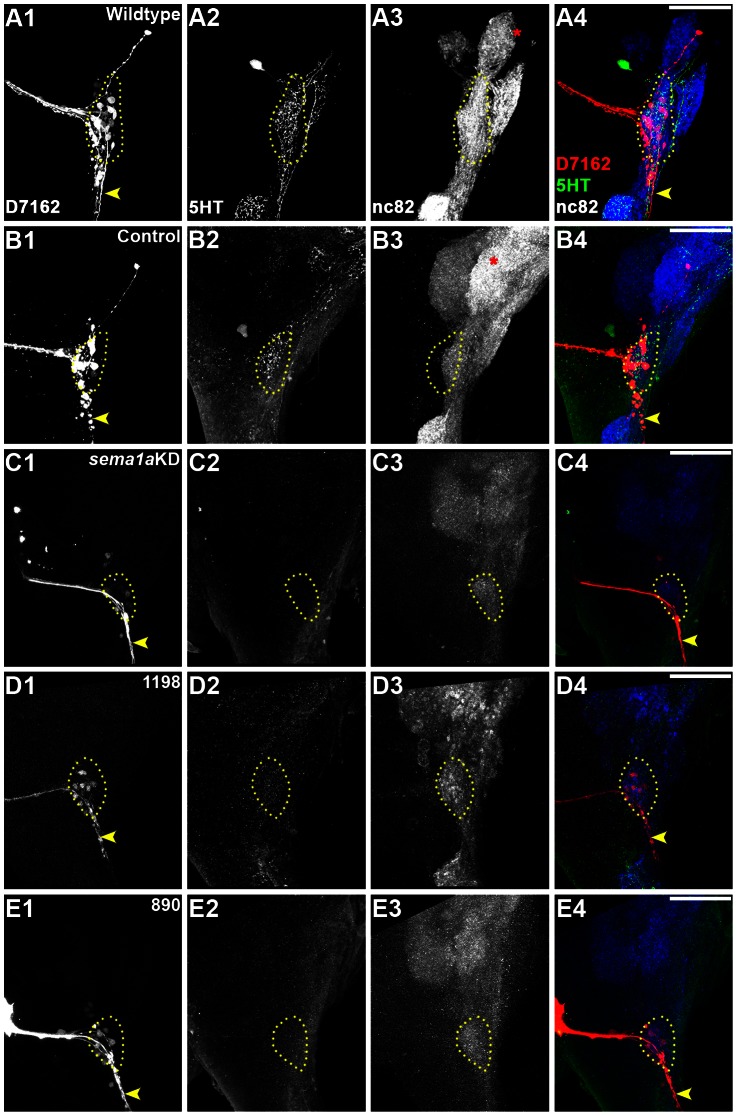
Larval antennal lobe defects in *sema1a* knockdown animals. In wildtype (A) and control-fed (B) L4 larvae, antennal sensory neurons innervate the antennal lobe (marked by dotted yellow circle throughout the figure), which is labeled by mAb nc82 staining (A3, B3). Serotonergic projection neurons are marked by 5HT staining in the antennal lobes of these animals (A2, B2; overlays of all three labels are shown in A4 and B4). *sema1a* knockdown animals (C–E) show a reduction in the number of antennal neurons (C1, D1, E1) targeting the antennal lobe, which is marked by reduced mAb nc82 synaptic neuropil staining (C3, D3, E3; weak levels are slightly increased in these panels so that the antennal lobe staining can be viewed). Reduced anti-serotonin 5HT staining of projection neurons is also observed in knockdown animals (C2, D2, E2; overlays of all three labels are shown in C4, D4, and E4). Comparable antennal lobe *sema1a* knockdown phenotypes are observed in animals fed with siRNA^1198^ (D), siRNA^890^ (E), or a combination of the two (*sema1a* KD in C). Dorsal is up in all panels. Scale bar = 25 µm.

A combination of anterograde labeling of antennal sensory neurons and histochemical marker analyses was used to examine antennal lobe development in L4 nanoparticle control-fed vs. *sema1a* knockdown animals. In chitosan/siRNA control-fed L4 animals, antennal sensory neuron tracts enter the developing antennal lobe region (Fig. 4B.1), which is marked through mAb nc82 co-staining (Fig. 4B.3). In addition to marking the antennal lobe, detection of the mAb nc82 label, a synaptic neuropil marker [28], suggested that the antennal sensory neurons had synapsed with antennal lobe projection neurons. Expression of serotonin (5HT), a projection neuron marker [28], was in fact detected in the antennal lobes of these animals (Fig. 4B.2). In these experiments, control-fed animals (Fig. 4B) resembled wildtype animals reared on a normal diet (Fig. 4A), suggesting that control chitosan/siRNA nanoparticle feeding itself did not disrupt antennal lobe development.

Anterograde labeling experiments demonstrated that fewer antennal sensory neurons innervate the developing antennal lobe in *sema1a* knockdown L4 animals (Fig. 4C.1). This reduction did not appear to result from death of sensory neurons, which still expressed appropriate markers in their cell bodies (data not shown). *sema1a* knockdown L4 larvae were also assessed through staining with mAb nc82 and anti-5HT antibodies. *sema1a* knockdown individuals display a decrease in overall expression of both markers within the antennal lobe region (Fig. 4C.2,3; compare to control-fed animals in Fig. 4B.2,3). These data correlated well with the reduced number of ORNs innervating the antennal lobe in these knockdown animals (Fig. 4C.1). Although antennal lobe defects were detected in 44% of *sema1a* knockdown animals (n = 35), antennal lobe defects were not observed in any control-fed animals (Fig. 4B; n = 69). Furthermore, all of the *sema1a* knockdown phenotypes described above were detected in knockdown larvae that were fed either a combination of siRNA^890^ and siRNA^1198^ (Fig. 4C) or animals that were fed with either individual siRNA alone (Fig. 4D,E). These results suggested that the observed phenotypes result from loss of *sema1a* expression and are not likely a result of off-site targeting by either siRNA. The cumulative results of these larval *sema1a* knockdown assays are summarized in Table 1.

**Table 1:** Quantification of antennal lobe defects in *sema1a* knockdown animals.

	Larvae		Pupae			
siRNA	n	Wildtype	AL defects	n	Wildtype	AL defects
**Control**	69	69 (100%)	0 (0%)	68	68 (100%)	0 (0%)
***sema1a*** ** KD**	77	42 (55%)	35 (44%)*	75	51 (68%)	24 (32%)*

The above set of data represents a compiled summary of results obtained for control-fed vs. *sema1a* knockdown (KD) animals from a total of eight replicate experiments performed in both larvae (left) and pupae (right). The total number of animals assessed (n) and numbers/percentages of animals displaying wildtype morphology vs. antennal lobe (AL) defects (olfactory sensory neuron targeting defects, lack of neuropil and glomeruli formation) are indicated. Knockdown was immunohistochemically confirmed through anti-Sema1a antibody staining in a subset of animals (15 larvae and 10 pupae) displaying the most severe defects (denoted with *). All KD animals assessed in this manner were found to have reduced levels of Sema1a, while Sema1a levels in control-fed animals were normal (examples are shown in Figures 2 and 7).

**Figure 5:**
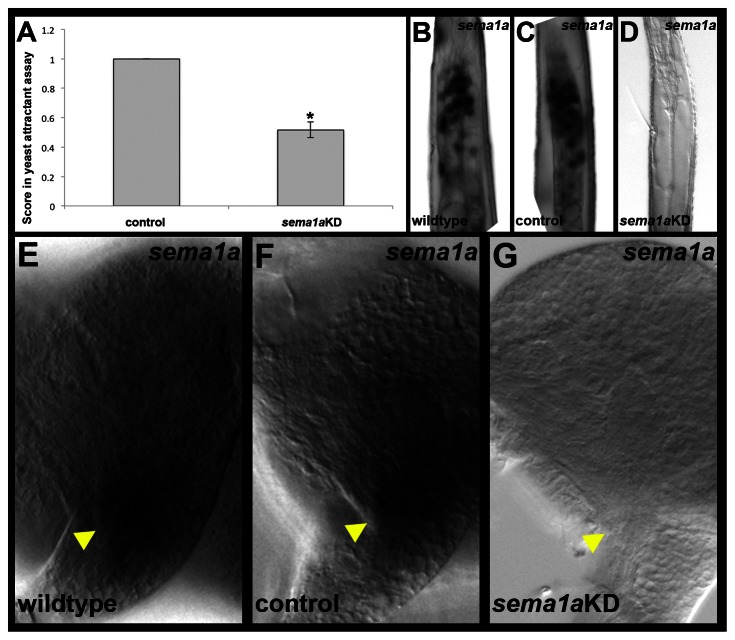
A decreased response to yeast odorant attractant is observed in *sema1a* knockdown animals. Control-fed and *sema1a* knockdown animals were assessed for their response to a yeast odorant attractant. In this assay, individual animals that were attracted to the yeast were awarded a score of 1, while animals that were not attracted to the yeast received a score of 0. Average scores for control (n = 86) vs. knockdown (n = 87) animals are plotted in A. The mean score for *sema1a* individuals (0.5) was significantly lower than that of control-fed (mean = 1) animals (p<0.001). Levels of *sema1a* were severely reduced in the antennae (D) and brains (G) of *sema1a* knockdown animals that failed to respond to the yeast; compare to *sema1a* transcript levels in control-fed antennae (C) and brains (F), which were similar to *sema1a* transcript levels found in wildtype animals (B,E). The proximal ends of antennae are oriented upward in B–D. Dorsal is oriented upward, and yellow arrowheads mark the antennal lobes in E–G.

Individual L4 larvae were next tested in an olfactory-driven behavioral assay adapted from previous designs [34]. Individual control-fed and *sema1a* knockdown larvae were assayed for attraction to a yeast odorant pellet. All control-fed individuals (n = 86) touched the yeast pellet during the assay (Fig. 5A, Table 2), and these animals were found to have wildtype levels of *sema1a* expression in their antennae and brains (Fig. 5C,F, compare to 5B,E; Table 2). Knockdown of *sema1a* resulted in significantly reduced performance in the yeast odorant attractant assay (p<0.001; Fig. 5A). Of the individuals that fed on *sema1a* knockdown nanoparticles (n = 87), 48% failed to touch the yeast pellet. Levels of *sema1a* were moderately or severely reduced in the antennae and brains (Fig. 5D,G) of all knockdown animals that failed to respond to the yeast (Table 2). Thus, decreased levels of *sema1a* correlated with poor performance in the behavioral assay. *sema1a* transcripts could still be detected in all knockdown nanoparticle-fed individuals that were attracted to the yeast (Table 2), suggesting that knockdown levels were not sufficient enough to impact their performance in the assay. Cumulatively, these results indicate that loss of *sema1a* decreases the L4 larval response to a yeast attractant.

**Table 2:** Levels of *sema1a* correlate with performance in yeast behavioral assays.

		Attracted				Not attracted			
siRNA	n	# Animals	Normal	Moderate	Null	# Animals	Normal	Moderate	Null
**Control**	86	86 (100%)	86(100%)	0	0	0	0	0	0
***sema1a*** ** KD**	87	45 (52%)	37 (82%)	8 (18%)	0	42 (48%)	0	11 (26%)	31(74%)

The above set of data represents a compiled summary of results obtained from four replicate experiments in which control-fed vs. *sema1a* knockdown (KD) animals were tested in a yeast odorant attractant assay. The total number of animals (n) indicates the number of individuals that were assessed in these assays. The number of individuals (# Animals) that were attracted (left; animals that touched the yeast pellet and received a score of 1) or not attracted (right; animals that did not touch the yeast pellet and received a score of 0) under each condition (Control or KD) are indicated, and the percentages of total animals are reported after the raw numbers. The levels of *sema1a* were assessed in the brains and antennae of animals attracted (left) or not attracted (right) to the yeast. The raw number/percentage of # animals with Normal (wildtype *sema1a*), Null (no detectable *sema1a* transcript), or Moderate (reduced but not wildtype *sema1a*) levels are indicated. Loss of *sema1a* expression correlated well with a lack of attraction to the yeast.

**Figure 6:**
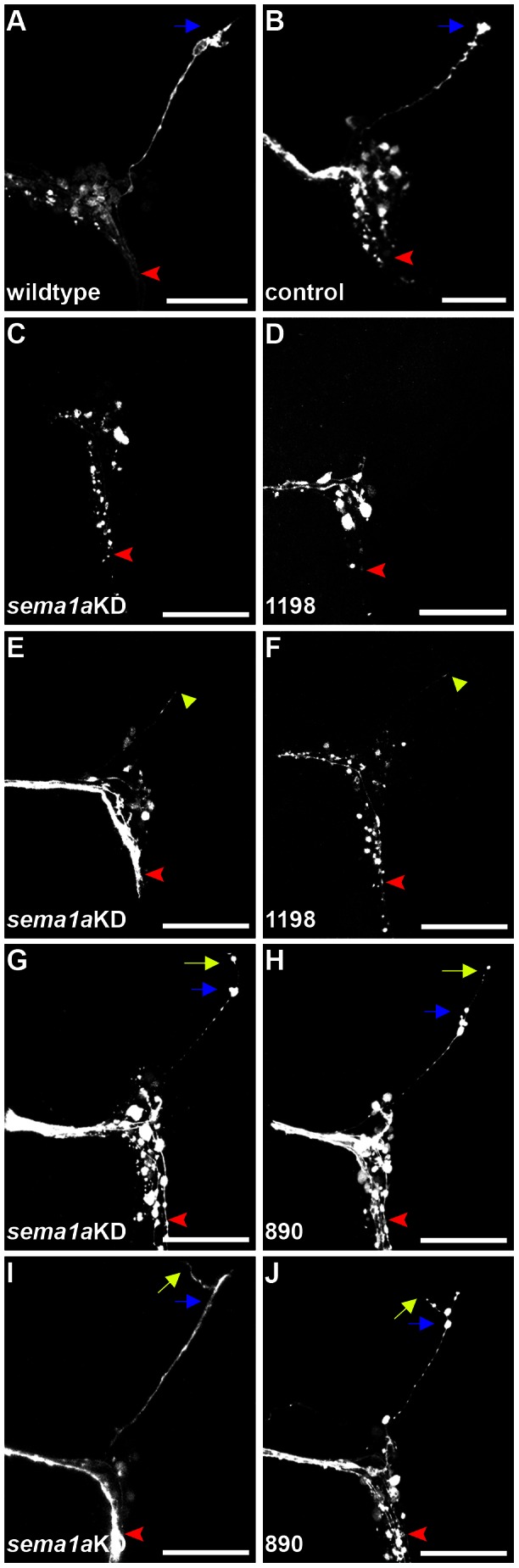
Larval antennal neuron targeting defects in *sema1a* knockdown animals. In the larval antennal lobe, antennal gustatory neurons (red arrowhead) target the antennal lobe and project ventrally (oriented down in all panels) toward the subesophageal ganglion. These neurons target properly in *sema1a* knockdown animals (C–J). In wildtype (A) and control-fed (B) L4 larvae, a subset of antennal sensory neurons innervating the antennal lobe send projection neurons dorsally (oriented up in all panels) to a target region (marked by blue arrows) in the SuEG. These neurons do not target properly to the SuEG in *sema1a* knockdown animals (C–J). Phenotypic categories observed include: antennal neurons failing to innervate outside of the antennal lobe (C, D; 38% of phenotypes; n = 38), neurons (yellow arrowheads) stopping short of the SuEG target (E, F; 19% of phenotypes), extension beyond the target region (blue arrows) within the SuEG (G, H; 24% of phenotypes), and extraneous branching within the SuEG (I, J; 19% of phenotypes). All four phenotypes were detected in animals fed with siRNA^890^ alone (H, J), siRNA^1198^ alone (D, F), or a combination of the two siRNAs (referred to as *sema1a* KD in C, E, G, and I), although only a subset of these results are included here. These phenotypes were never observed in wildtype larvae (A) or those that were fed with control siRNA nanoparticles (B). Scale bar = 25 µm.

This behavioral difference correlates well with the olfactory defects noted in *sema1a* knockdown animals (Fig. 4C–F). However, given the complexity of feeding behavior, it is possible that the *sema1a* behavioral phenotype could result at least in part from other defects in *sema1a* animals. Neither the control nor *sema1a* knockdown nanoparticle-fed animals displayed any obvious locomotor defects, indicating that locomotor deficits did not appear to contribute to the behavioral defect. It is also possible that the reduced attraction could result from gustatory defects. In the larval antennal lobe, a subset of antennal sensory neurons project ventrally from the lobe to the subesophageal ganglion (Figs. 4A.1, 6A). Based on studies in other insects, these neurons are likely to be gustatory neurons [23], [35], [36]. No defects in this subset of neurons were noted in *sema1a* knockdown L4 larvae (Figs. 4C.1, D.1, E.1, 6C–J vs. control-fed in animals in Figs. 4B.1, 6B), suggesting that larval antennal gustatory neurons target properly in *sema1a* knockdown animals. It was noted that a second subset of antennal sensory neurons which project dorsally from the larval antennal lobe to a region in the supraesophageal ganglion (SuEG) were disrupted in L4 *sema1a* knockdown animals (Fig. 6C–J). To our knowledge, the function of these neurons is unknown, but documentation of these phenotypes is nevertheless provided. In summary, these analyses uncovered L4 antennal lobe ORN defects that correlate well with a decreased attraction to yeast behavioral phenotype, but neither gustatory nor locomotor defects were noted in *sema1a* knockdown L4 animals.

### *sema1a* is critical for neuronal targeting in the late stages of *Ae. aegypti* olfactory development

**Figure 7:**
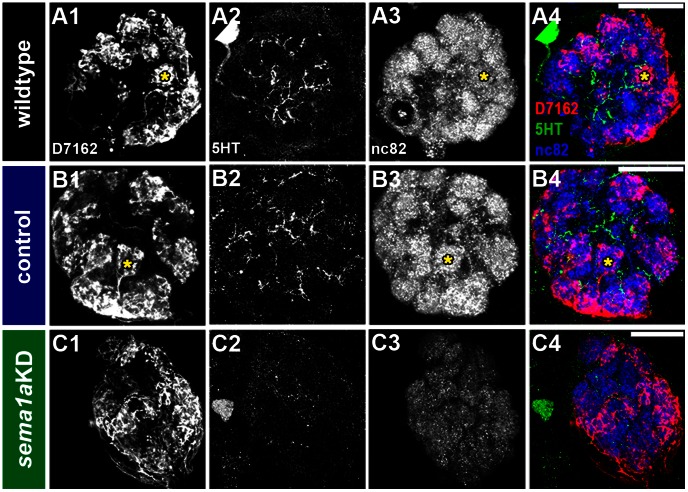
*sema1a* knockdown disrupts pupal antennal ORN targeting. In wildtype (A1–A4) and control-fed (B1–B4) pupae at 24 hours APF, ORNs innervate the antennal lobe and target specific glomeruli within the lobe (dye fills in A1, B1; mAb nc82 staining in A3, B3). Distinct glomerular structures are fully formed by this time point (asterisks in A, B). *sema1a* knockdown animals show defects in ORN targeting and glomerular structure (dye fills in C1, mAb nc82 label in C3). Levels of both serotonergic neuronal (C2) and synaptic neuropil markers (C3) were decreased in *sema1a* knockdown animals as compared to wildtype (A2, A3) and control-fed (B2, B3) larvae. Levels of mAb nc82 staining were increased slightly in C3 so that morphological phenotypes are viewable. Dorsal is up in all panels, and overlays of all three labels are depicted at right. Comparable sections of the antennal lobe are shown in panels A, B, and C. Panels A4, B4 & C4 represent the merged images from their respective first three columns. Scale bar = 25 µm.

**Figure 8:**
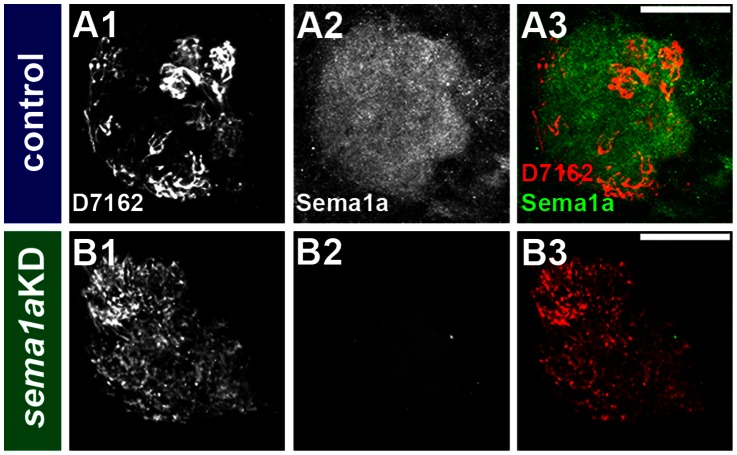
Pupal ORN targeting defects correlate with *sema1a* knockdown. Severe ORN targeting defects (dye fills in B1; compare to control-fed animal in A1) correlate with loss of Sema1a protein expression at 24 hours APF (detected through lack of antibody staining in B2; compare to control-fed in A2). Overlays are shown in A3 and B3.

We next examined if olfactory phenotypes could be detected in *sema1a* knockdown animals during the pupal stage, a time point that more closely resembles the morphology of the adult olfactory system [28]. Anterograde labeling was used to trace neurons from the antennae to the developing antennal lobes in control-fed and *sema1a* knockdown pupae. At 24 hours APF in wildtype (Fig. 7A.1) and control-fed pupae (Fig. 7B.1), ORNs innervate the antennal lobe and target specific glomeruli within the lobe. In *sema1a* knockdown individuals, ORNs fail to sort into individual glomeruli (Fig. 7C.1). Decreased serotonin (Fig. 7C.2) and mAb nc82 staining levels were also noted (Fig. 7C.3; compare to control-fed in Fig. 7B.2, 3). These results suggested that the synaptic neuropil had not formed properly in these animals, in which a collapse in the structure and shape of the antennal lobe and individual glomeruli as compared to control individuals was noted (Fig. 7C.3; compare to 7B.3). These phenotypes directly correlated to *sema1a* knockdown (Fig. 8B). Although 32% of *sema1a* knockdown pupae (n = 24) displayed antennal lobe defects (Fig. 7C), no such defects were detected in control-fed animals (n = 51; Fig. 7B), which resembled wildtype pupae reared on a normal diet (Fig. 7A). Furthermore, as with the larval experiments (Figs. 4, 6) the detection of antennal lobe defects in *sema1a* knockdown pupae fed either siRNA^890^, siRNA^1198^ (not shown), or a combination of the two (Fig. 7C) suggested that the pupal antennal lobe phenotypes detected were not likely a result of off-site targeting by either siRNA. The cumulative results of these pupal *sema1a* knockdown assays are summarized in Table 1.

## Discussion

### Analysis of vector mosquito olfactory system development

We recently began to address the need for developmental research in mosquitoes through the pursuit of functional developmental genetic studies in *Ae. aegypti* [1]. Our work led to the publication of methodology [25], [27], [31], [37], [38] that was utilized to study gene function in the developing embryonic nerve cord, in which Sema1a regulates axon guidance [20]. These investigations represented to our knowledge the first reported targeted knockdowns of embryonic genes in any vector mosquito species. In this investigation, analysis of *Aae sema1a* was extended to the larval and pupal stages, in which its function was characterized during olfactory development. The genetics of olfactory system development has not been functionally assessed in insects other than *D. melanogaster*. The ability of insects to adapt to their many environments is exhibited in a wealth of morphological and functional adaptations in their peripheral sensory organs and central nervous systems [39]. To date, the exploration of genetic mechanisms underlying this diversity has been hindered by a lack of functional genetic analyses of arthropod nervous system development outside of *D. melanogaster* [39]. In the case of vector mosquitoes, which are responsible for some of the worst scourges of humankind, exploring neural diversity is not only of academic interest, but it is also of medical and socioeconomic importance [40]. This investigation has paved the way for further analysis of developmental genes that regulate olfactory system development in *Ae. aegypti*. Moreover, this study advocates the broader use of chitosan/siRNA nanoparticles to characterize post-embryonic developmental gene function in mosquitoes and potentially other arthropods.

### A requirement for Sema1a in the developing *Ae. aegypti* larval antennal lobe

Although pupal olfactory phenotypes are known to result from loss of *sema1a* in *D. melanogaster* [13]–[15], this study is the first to our knowledge to document a requirement for Sema1a during insect larval antennal lobe development. Insect larval nervous system developmental genetics has not been extensively characterized outside of *D. melanogaster*. As observed in *D. melanogaster* [23], the *Ae. aegypti* larval olfactory system mimics the architecture of the adult, but is reduced in cell number (Figs. 1A,B, 3A,B, 4A, 6A). This reduced complexity facilitated detailed examination of olfactory development in wildtype and *Aae sema1a* knockdown animals. Knockdown of *sema1a* resulted in a variety of larval antennal lobe defects, including a decrease in the number of neurons targeting the antennal lobe (Fig. 4C.1–E.1). Reduced staining for serotonin (projection neuron marker; Fig. 4C.2–E.2) and also mAb nc82, a marker of the synaptic neuropil (Fig. 4C.3–E.3) was noted. These results are likely indicative of a reduction in the number of synapses found in the antennal lobe of *sema1a* knockdown larvae.

These severe *sema1a* knockdown antennal lobe phenotypes (Fig. 4C–E) correlated with a yeast attractant behavioral defect (Fig. 5, Table 2). It is of course difficult to be absolutely certain that the antennal lobe defects in *sema1a* knockdown animals (Fig. 4C–E), which are fairly extensive, are the only contributor to the behavioral phenotype observed. Although our screening of gustatory neuron targeting and locomotor function assessment did not uncover any such defects in *sema1a* knockdown animals (Figs. 4C.1–E.1, 6C–J), feeding behavior is complicated, and Sema1a expression is detected in other portions of the brain (Fig. 1A.2, B.2), including the optic lobe (Mysore et al., in preparation), that may also contribute to the *sema1a* knockdown larvae's decreased attraction to yeast. Still, the results of these behavioral assays correlate well with the severe antennal lobe defects observed in *sema1a* knockdown animals (Fig. 4).

### Sema1a is required for glomeruli formation in *Ae. aegypti*

Genetic mosaic analyses in *D. melanogaster* demonstrated that Sema1a functions nonautonomously to ensure that pupal ORNs target to glomeruli [14], [15]. Although we of course lack the genetic tools to perform comparable mosaic analyses in *Ae. aegypti*, whole organism siRNA knockdown experiments show the effects of a more broad scale knockdown of *Aae sema1a* on antennal lobe formation (Figs. 2E, 7C, 8B), something which is not assessed in mosaic analyses. These knockdown experiments demonstrated that *Aae* Sema1a is required for sorting of ORNs into specific glomeruli (Fig. 7C.1, 8B.1). Interestingly the phenotypes observed in the *Ae. aegypti* pupal stages, including a lack of distinct glomeruli structure formation (Figs. 2E.2, 7C, 8B), are consistent with the model proposed for *D. melanogaster* Sema1a function [15]. Previous work [14], [15] suggested that Sema1a acts non-cell autonomously as a repellant to regulate sorting of different classes of ORNs expressing various OR proteins, and thereby promoting the segregation and formation of protoglomeruli. Due to the lack of comparable genetic tools in mosquitoes, one cannot adequately address whether *Aae* Sema1a functions cell autonomously or non-cell autonomously. However, comparable to *D. melanogaster*, differential Sema1a expression is detected among neighboring glomeruli (Figs. 1C.3, 2D.1). As discussed by Latteman *et al.* [15], this differential Sema1a expression pattern may provide local positional information during class-specific axonal convergence.

Komiyama *et al.* [13] demonstrated that graded levels of Sema1a expression in the antennal lobe of *D. melanogaster* are responsible for proper projection neuron dendritic targeting into the antennal lobe, which is critical if odorant information is to be properly relayed to higher order brain structures. Dendritic targeting could not be properly assessed in the *Ae. aegypti* antennal lobe due to technical limitations. However, loss of *Aae sema1a* resulted in a decrease in 5HT-labeling of projection neurons (Fig. 7C.2) and mAb nc82-labeling of the synaptic neuropil (Fig. 7C.3), suggesting that the number of synapses in the pupal antennal lobe is reduced. Furthermore, as discussed above, a graded pattern of Sema1a expression is detected in the *Ae. aegypti* pupal antennal lobe (Figs. 1C.3, 2D.1), and it is therefore possible that the function of Sema1a in dendritic targeting is conserved between *D. melanogaster* and *Ae. aegypti*. Moreover, Sweeney *et al.* [14] demonstrated that degenerating larval ORNs direct adult projection neuron dendrite targeting. It is therefore possible that the defects observed in ORNs of *sema1a* knockdown larvae (Figs. 4C–E) may also impact projection neuron dendritic targeting at later stages of olfactory development in *Ae. aegypti*.

### siRNA chitosan/nanoparticle gene targeting in *Ae. aegypti*

This study demonstrates, to our knowledge for the first time, that chitosan/siRNA nanoparticle-mediated gene targeting can be used to disrupt olfactory system development in insects. We had previously used microinjection as a means of delivering custom-designed siRNAs into *Ae. aegypti* embryos [18], [20]. However, embryonic microinjections are not an effective means of assessing the larval functions of genes that are lethal when targeted in embryos. Microinjection of larvae and pupae can be used to deliver siRNAs, and we found that microinjection of *sema1a* knockdown siRNAs did in fact generate significant knockdown of *sema1a* transcripts in L4 larvae (not shown). However, we ultimately opted to pursue knockdown through chitosan/siRNA nanoparticle feeding, which is technically less challenging, allowed for the inclusion of more animals per replicate, and could also potentially be implemented in the field. Furthermore, the nanoparticle knockdown strategy is less stressful to the organism than microinjection, a particular advantage when behavioral assays follow knockdown experiments, as was the case in this investigation.

The average *sema1a* knockdown levels generated through chitosan/siRNA nanoparticle feeding in *Ae. aegypti* are in line with those reported by Zhang *et al.* [26], who used this strategy to knockdown chitin synthase genes in *An. gambiae*. In this investigation, *sema1a* expression levels were examined in individual animals through *in situ* hybridization and protein staining. These studies demonstrated that within the population of knockdown animals, one finds individuals with nearly complete loss of *sema1a* (Figs. 2C, E.1, 3D, Fig. 5D, G, 8B) in the developing olfactory system. Thus, use of chitosan/siRNA nanoparticles allowed for generation of the equivalent of a *sema1a* conditional null mutant in *Ae. aegypti*. Furthermore, co-staining for Sema1a expression allowed us to identify and score phenotypes specifically in these animals with the greatest levels of knockdown. This greatly simplified phenotype analyses which can be complicated by the variable levels of knockdown that inevitably accompany whole organism RNAi experiments. Moreover, these experiments demonstrated that the central and sensory nervous systems are not refractory to siRNAs. Thus, chitosan siRNA nanoparticle gene targeting is a useful tool for analysis of olfactory system development in *Ae. aegypti*, and it will be useful to study knockdown efficiency with respect to additional gene targets. It is likely that this technology could be implemented to study olfactory system development in additional arthropod species, and it would be interesting to investigate the relative effectiveness of this knockdown strategy in a variety of different arthropod tissue types. It may also be possible to improve upon the existing nanoparticle RNAi methodology (for example by modifying the feeding strategy, using different types of nanoparticles, or different types of siRNAs) to increase knockdown efficiency.

As suggested by Zhang *et al.* [26], chitosan-based dsRNA nanoparticles could be used for pest control. Chitosan nanoparticles are non-toxic to insects and appear to require nothing more than particle ingestion to target genes of interest in mosquitoes. The results of our investigation build upon their observations by using chitosan/nanoparticle RNAi to disrupt development of the mosquito olfactory system, a tissue of vector importance, and also by extending this methodology to another vector mosquito species, *Ae. aegypti*. Moreover, in our investigation, nanoparticles were used to deliver siRNAs as opposed to longer pieces of dsRNA. Custom siRNAs can be produced commercially in mass, which would facilitate their use in integrated vector management strategies if obstacles such as cost and delivery could be addressed. Also, given the short length of siRNAs, it is easier to design them to be both gene and species-specific, which decreases the chances of off-site targeting, an advantage that is helpful both at the bench and perhaps even one day, in the field.

## References

[1] Clemons A, Haugen M, Flannery E, Tomchaney M, Kast K, et al (2010) *Aedes aegypti:* an emerging model for vector mosquito development. Cold Spring Harb Protoc 2010: pdb emo141. 20889691 10.1101/pdb.emo141 PMC2975269

[2] Hallem EA, Dahanukar A, Carlson JR (2006) Insect odor and taste receptors. Annu Rev Entomol 51: 113–135. 16332206 10.1146/annurev.ento.51.051705.113646

[3] Raminani LN, Cupp EW (1978) Embryology of *Aedes aegypti* (L.) (Diptera: Culicidae): organogenesis. Int J Insect Morphol & Embryol 7: 273–296.

[4] Holt RA, Subramanian GM, Halpern A, Sutton GG, Charlab R, et al (2002) The genome sequence of the malaria mosquito *Anopheles gambiae* . Science 298: 129–149. 12364791 10.1126/science.1076181

[5] Nene V, Wortman JR, Lawson D, Haas B, Kodira C, et al (2007) Genome sequence of *Aedes aegypti*, a major arbovirus vector. Science 316: 1718–1723. 17510324 10.1126/science.1138878 PMC2868357

[6] Arensburger P, Megy K, Waterhouse RM, Abrudan J, Amedeo P, et al (2010) Sequencing of *Culex quinquefasciatus* establishes a platform for mosquito comparative genomics. Science 330: 86–88. 20929810 10.1126/science.1191864 PMC3740384

[7] Bohbot JD, Dickens JC (2009) Characterization of an enantioselective odorant receptor in the yellow fever mosquito *Aedes aegypti* . PLoS One 4: e7032. 19753115 10.1371/journal.pone.0007032 PMC2737144

[8] Bohbot JD, Fu L, Le TC, Chauhan KR, Cantrell CL, et al (2011) Multiple activities of insect repellents on odorant receptors in mosquitoes. Med Vet Entomol 25: 436–444. 21395633 10.1111/j.1365-2915.2011.00949.x

[9] Bohbot JD, Jones PL, Wang G, Pitts RJ, Pask GM, et al (2011) Conservation of indole responsive odorant receptors in mosquitoes reveals an ancient olfactory trait. Chem Senses 36: 149–160. 20956733 10.1093/chemse/bjq105 PMC3020388

[10] Grant AJ, Dickens JC (2011) Functional characterization of the octenol receptor neuron on the maxillary palps of the yellow fever mosquito, *Aedes aegypti* . PLoS One 6: e21785. 21738794 10.1371/journal.pone.0021785 PMC3128099

[11] Flannery E, Duman-Scheel M (2009) Semaphorins at the interface of development and cancer. Curr Drug Targets 10: 611–619. 19601765 10.2174/138945009788680383

[12] Kolodkin AL, Matthes DJ, Goodman CS (1993) The semaphorin genes encode a family of transmembrane and secreted growth cone guidance molecules. Cell 75: 1389–1399. 8269517 10.1016/0092-8674(93)90625-z

[13] Komiyama T, Sweeney LB, Schuldiner O, Garcia KC, Luo L (2007) Graded expression of semaphorin-1a cell-autonomously directs dendritic targeting of olfactory projection neurons. Cell 128: 399–410. 17254975 10.1016/j.cell.2006.12.028

[14] Sweeney LB, Couto A, Chou YH, Berdnik D, Dickson BJ, et al (2007) Temporal target restriction of olfactory receptor neurons by Semaphorin-1a/PlexinA-mediated axon-axon interactions. Neuron 53: 185–200. 17224402 10.1016/j.neuron.2006.12.022

[15] Lattemann M, Zierau A, Schulte C, Seidl S, Kuhlmann B, et al (2007) Semaphorin-1a controls receptor neuron-specific axonal convergence in the primary olfactory center of *Drosophila* . Neuron 53: 169–184. 17224401 10.1016/j.neuron.2006.12.024

[16] Bashaw GJ (2007) Semaphorin directs axon traffic in the fly olfactory system. Neuron 53: 157–159. 17224397 10.1016/j.neuron.2007.01.002

[17] Eickhoff R, Bicker G (2012) Developmental expression of cell recognition molecules in the mushroom body and antennal lobe of the locust *Locusta migratoria* . J Comp Neurol 520: 2021–2040. 22173776 10.1002/cne.23026

[18] Clemons A, Haugen M, Le C, Mori A, Tomchaney M, et al (2011) siRNA-mediated gene targeting in *Aedes aegypti* embryos reveals that frazzled regulates vector mosquito CNS development. PLoS One 6: e16730. 21304954 10.1371/journal.pone.0016730 PMC3031613

[19] Behura SK, Haugen M, Flannery E, Sarro J, Tessier CR, et al (2011) Comparative genomic analysis of *Drosophila melanogaster* and vector mosquito developmental genes. PLoS One 6: e21504. 21754989 10.1371/journal.pone.0021504 PMC3130749

[20] Haugen M, Flannery E, Tomchaney M, Mori A, Behura SK, et al (2011) Semaphorin-1a is required for *Aedes aegypti* embryonic nerve cord development. PLoS One 6: e21694. 21738767 10.1371/journal.pone.0021694 PMC3124551

[21] Rodrigues V, Hummel T (2008) Development of the *Drosophila* olfactory system. Adv Exp Med Biol 628: 82–101. 18683640 10.1007/978-0-387-78261-4_6

[22] Brochtrup A, Hummel T (2011) Olfactory map formation in the *Drosophila* brain: genetic specificity and neuronal variability. Curr Opin Neurobiol 21: 85–92. 21112768 10.1016/j.conb.2010.11.001

[23] Gerber B, Stocker RF (2007) The *Drosophila* larva as a model for studying chemosensation and chemosensory learning: a review. Chem Senses 32: 65–89. 17071942 10.1093/chemse/bjl030

[24] Xia Y, Wang G, Buscariollo D, Pitts RJ, Wenger H, et al (2008) The molecular and cellular basis of olfactory-driven behavior in *Anopheles gambiae* larvae. Proc Natl Acad Sci U S A 105: 6433–6438. 18427108 10.1073/pnas.0801007105 PMC2359781

[25] Clemons A, Mori A, Haugen M, Severson DW, Duman-Scheel M (2010) Culturing and egg collection of *Aedes aegypti* . Cold Spring Harb Protoc 2010: pdb prot5507. 20889704 10.1101/pdb.prot5507 PMC2966317

[26] Zhang X, Zhang J, Zhu KY (2010) Chitosan/double-stranded RNA nanoparticle-mediated RNA interference to silence chitin synthase genes through larval feeding in the African malaria mosquito *(Anopheles gambiae)* . Insect Mol Biol 19: 683–693. 20629775 10.1111/j.1365-2583.2010.01029.x

[27] Clemons A, Flannery E, Kast K, Severson D, Duman-Scheel M (2010) Immunohistochemical analysis of protein expression during *Aedes aegypti* development. Cold Spring Harb Protoc 2010: pdb prot5510. 20889707 10.1101/pdb.prot5510 PMC2976535

[28] Mysore K, Flister S, Muller P, Rodrigues V, Reichert H (2011) Brain development in the yellow fever mosquito *Aedes aegypti:* a comparative immunocytochemical analysis using cross-reacting antibodies from *Drosophila melanogaster* . Dev Genes Evol 221: 281–296. 21956584 10.1007/s00427-011-0376-2

[29] Yu HH, Araj HH, Ralls SA, Kolodkin AL (1998) The transmembrane Semaphorin Sema I is required in *Drosophila* for embryonic motor and CNS axon guidance. Neuron 20: 207–220. 9491983 10.1016/s0896-6273(00)80450-x

[30] Patel N (1996) *In situ* hybridization to whole mount *Drosophila* embryos. Krieg PA, editor. New York: Wiley-Liss. 357–370 p.

[31] Haugen M, Tomchaney M, Kast K, Flannery E, Clemons A, et al (2010) Whole-mount *in situ* hybridization for analysis of gene expression during *Aedes aegypti* development. Cold Spring Harbor Protocols 2010: pdb.prot5509. 20889706 10.1101/pdb.prot5509 PMC3076929

[32] Mysore K, Shyamala BV, Rodrigues V (2010) Morphological and developmental analysis of peripheral antennal chemosensory sensilla and central olfactory glomeruli in worker castes of *Camponotus compressus* (Fabricius, 1787). Arthropod Struct Dev 39: 310–321. 20438861 10.1016/j.asd.2010.04.003

[33] Nakanishi A, Nishino H, Watanabe H, Yokohari F, Nishikawa M (2010) Sex-specific antennal sensory system in the ant *Camponotus japonicus:* glomerular organizations of antennal lobes. J Comp Neurol 518: 2186–2201. 20437523 10.1002/cne.22326

[34] Liu C, Pitts RJ, Bohbot JD, Jones PL, Wang G, et al (2010) Distinct olfactory signaling mechanisms in the malaria vector mosquito *Anopheles gambiae* . PLoS Biol 8: pii: e1000467. 20824161 10.1371/journal.pbio.1000467 PMC2930861

[35] Heimbeck G, Bugnon V, Gendre N, Häberlin C, Stocke RF (1999) Smell and taste perception in *Drosophila melanogaster* larva: toxin expression studies in chemosensory neurons. Neuroscience 19: 6599–6609. 10414987 10.1523/JNEUROSCI.19-15-06599.1999 PMC6782832

[36] Ignell RH, Hansson BS (2005) Projection patterns of gustatory neurons in the suboesophageal ganglion and tritocerebrum of mosquitoes. The Journal of Comparative Neurology 492: 214–233. 16196031 10.1002/cne.20691

[37] Clemons A, Haugen M, Flannery E, Kast K, Jacowski C, et al (2010) Fixation and preparation of developing tissues from *Aedes aegypti* . Cold Spring Harb Protoc 2010: pdb prot5508. 20889705 10.1101/pdb.prot5508 PMC2976529

[38] Clemons A, Haugen M, Severson D, Duman-Scheel M (2010) Functional analysis of genes in *Aedes aegypti* embryos. Cold Spring Harb Protoc 2010: pdb prot5511. 20889708 10.1101/pdb.prot5511 PMC2957646

[39] Hansson BS, Stensmyr MC (2011) Evolution of insect olfaction. Neuron 72: 698–711. 22153368 10.1016/j.neuron.2011.11.003

[40] Manguin S, Boëte C (2011) Global impact of mosquito biodiversity, human vector-borne diseases and environmental change. In: Pujol JL, editor. The Importance of Biological Interactions in the Study of Biodiversity. InTech.

